# Super-Cooling Point and Effects of Temperature, Moisture, and Habitat on Overwintering Survival of the Threatened American Burying Beetle *Nicrophorus americanus*

**DOI:** 10.3390/biology15161417

**Published:** 2026-08-18

**Authors:** William Wyatt Hoback, Amelea Jones, Leonardo Vieira Santos, Rafael Hayashida, Michael Cavallaro

**Affiliations:** Department of Entomology and Plant Pathology, Oklahoma State University, Stillwater, OK 74078, USA; amelea.jones@okstate.edu (A.J.); lvieirasantos95@gmail.com (L.V.S.); rafael.hayashida@okstate.edu (R.H.); michael.cavallaro@okstate.edu (M.C.)

**Keywords:** insect conservation, climate change, temperate overwintering, sexton beetles

## Abstract

Most species within the genus *Nicrophorus* (Staphylinidae: Silphinae: Necrophorini) overwinter as adults during cold months. However, species that were once widely distributed, such as the American burying beetle, *Nicrophorus americanus* Olivier, experience different overwintering conditions, altered by increasing climate variability, making cold periods less predictable. In this study, we determined the mean super-cooling point of Oklahoma *N. americanus* to be −1.98 °C, a value that suggests that beetles rely on behavioral avoidance of freezing soils rather than on physiological freeze tolerance or super-cooling. We conducted a series of field experiments in Oklahoma to test the effects of habitat and burial depth on the overwintering survival of *N. americanus*. Correlations were found between burial depth and the survival of beetles. Highly fluctuating winter temperatures and periods without precipitation increased mortality rates, with adults emerging from overwintering much earlier than previously documented. As climates become warmer and more variable, with temperature fluctuations and altered precipitation patterns, populations of the federally designated threatened *N. americanus* are at risk of extirpation.

## Simple Summary

The American burying beetle (*Nicrophorus americanus*) survives winter as an adult by burying itself the soil, where it remains for up to eight months in northern latitudes and four months in its southern range. While underground, the beetles must conserve metabolic energy, but the mechanisms of survival, along with how differences in overwintering habitat affect survival, are currently unknown. We measured the temperature at which beetles freeze to death and found the super-cooling point to be close to 0 °C, indicating that they avoid freezing or lower the freezing point of their bodies. Field studies showed that winter survival is associated with habitat. Beetles that could bury deeper had higher survival rates, but long winter dry periods greatly increased mortality, regardless of maximum burial depth. These results suggest that warmer winters and more variation in the frequency of precipitation jeopardizes the long-term survival of this federally designated threatened species.

## 1. Introduction

Many insects from temperate climates endure an overwintering phase for a significant part of their life cycles [1]. As poikilothermic organisms, they must avoid tissue damage in sub-zero temperatures to survive the winter period [2]. Factors such as an insect’s cold hardiness, temperature extremes and periodicity of fluctuation, duration of exposure, the presence of chill injury, and the developmental stage can determine the likelihood of survival [3,4,5]. Invertebrates in northern regions frequently exhibit physiological and behavioral adaptations to improve survival during periods of sub-zero temperatures [6]. Often, these insects enter diapause and exhibit freeze avoidance, lowering the freezing point of their hemolymph with salts or glycerol and creating a super-cooling point that is below 0 °C [7]. The super-cooling point of these insects can be further affected by their water content and degree of desiccation [4,8]. The super-cooling ability declines as body fluid volume rises, and active insects carry a comparatively high water content, given their small body size [9].

During cold periods, the insects experience lowered metabolic rates, as temperature-driven declines in metabolic function occur with colder temperatures [1,10]. Alternatively, some temperate insects exhibit freeze tolerance [11]. In freeze-avoidant insects, body fluid must remain unfrozen for survival, whereas freeze-tolerant insects use ice-nucleating proteins to control the timing and location of ice formation within their tissues [6,11]. These freeze-tolerant species can be further separated by their super-cooling point and lower lethal temperature [12]. Insect overwintering strategies are usually correlated with climate, with insects from the Northern Hemisphere more frequently exhibiting freeze tolerance and those from the Southern Hemisphere more commonly exhibiting freeze avoidance [11]. This pattern is generally attributed to the greater land masses of northern regions, which result in more extreme and predictable winter temperatures, whereas the smaller land masses and stronger oceanic influences of the Southern Hemisphere moderate temperature extremes [11].

A third insect overwintering strategy is to avoid exposure to extreme cold. Species using this strategy can bury vertically into the soil or seek thermal refugia. Because these insects remain inactive, predation pressure and the risk of starvation drop sharply during winter [1,10]. However, limited mobility becomes costly when environmental conditions deteriorate, as occurs during drought or flooding [1,13]. For species that overwinter belowground, soil properties strongly influence overwintering success [1]. Soil buffers temperature relative to the surface, and the strength of that buffering depends on burial depth, soil texture, moisture, and snow cover [1,14]. Insects tend to burrow deep enough to reach warm and stable temperatures, but probably no deeper, since digging is energetically expensive, and stored energy must last until activity resumes with warmer temperatures [1]. Deeper burial will also expose these insects to warmer temperatures, likely depleting their overwintering energy stores more rapidly. However, relatively few studies have presented data on burial depth or examined the effects of warming climates on temperate insect overwintering. Burying beetles in the genus *Nicrophorus* Fabricius, 1775 present a unique opportunity to study overwintering phenomena.

Burying beetles are temperate insects that typically breed in summer, with most species overwintering as adults [15]. For these species, adults can choose overwintering sites and potentially utilize carrion as a resource [16]. Beetles overwintering without a food source rely on accumulated fat until activity resumes. Because metabolism rate follows ambient temperature, warming and thermal variability both accelerate depletion of fat reserves [17]. Additionally, burying beetles are very sensitive to desiccation [18] and submersion [19], and changes in climate-driven moisture patterns may also limit survival for aestivating individuals. However, few studies have examined overwintering survival or response to environmental conditions, even for widespread species.

The American burying beetle *N. americanus* is the largest species native to North America. It once occurred throughout the eastern United States, occurring in 35 states and ranging from Canada to Florida and west to Nebraska and Oklahoma. It now has limited distribution at the edges of its former range, occurring with three separated populations concentrated in Nebraska extending into South Dakota, Oklahoma extending into Kansas and Arkansas, and on a small island off the coast of Rhode Island [20]. Based on its historic range, the species is classified as a habitat generalist with populations occurring in forest and prairie ecosystems [20]. The species overwinters as adults that were produced during the previous summer. Emerging adults seek food and a reproductive resource in the form of a suitable carcass weighing between 100 and 200 g [18]. Usually, the largest male and female defeat other burying beetles and work together to bury the suitable breeding resource and provide bi-parental care to raise an average of 15 offspring. Adults live approximately 100 days at warmer temperatures and are believed to be single-brooded [16,18,20,21]. Because of a dramatic decline in its range, the *N. americanus* was federally protected as an endangered species in 1989. With additional populations and greater numbers discovered since its listing, the species was reclassified as threatened in 2020 [22].

Understanding life history traits across the remaining range is important for the conservation of this species. In an Arkansas field trial, Schnell et al. [16] recorded an about 60% overwinter survival rate overall, with provisioned beetles surviving at a higher rate (77%) than non-provisioned beetles (45%). However, the experiments were conducted in the southern portion of the range, where soil temperatures stayed above freezing for the whole trial [16]. In Nebraska, the roundneck sexton beetle *Nicrophorus orbicollis* Say, 182 were buried below the frost line, up to 56 cm in depth, and appeared to follow the frost line, as they were found at shallower depths in warmer months [23].

Habitat-level variation in temperature is known to affect survival in other insects [24,25] and may impact survival of *N. americanus*. Grasslands and other sparsely vegetated sites offer less thermal buffering, so warmer winters may increase metabolic rates and decrease survival compared with the rates for forested areas comprising organic material and layers of leaf litter [25]. Additionally, bedrock depth could limit the ability of individuals to dig deeper during very cold temperatures and thus influence survival. We conducted a series of studies to determine the effects of habitat and limitations to burial depth on overwintering survival of *N. americanus*. Additionally, we determined the super-cooling point (SCP) of *N. americanus* adults. The SCP is the point at which an insect’s fluids freeze, releasing the latent heat of crystallization [6]. Because intracellular ice is damaging, individuals die immediately or soon after thawing.

## 2. Materials and Methods

### 2.1. Study Overview

**Figure 1:**
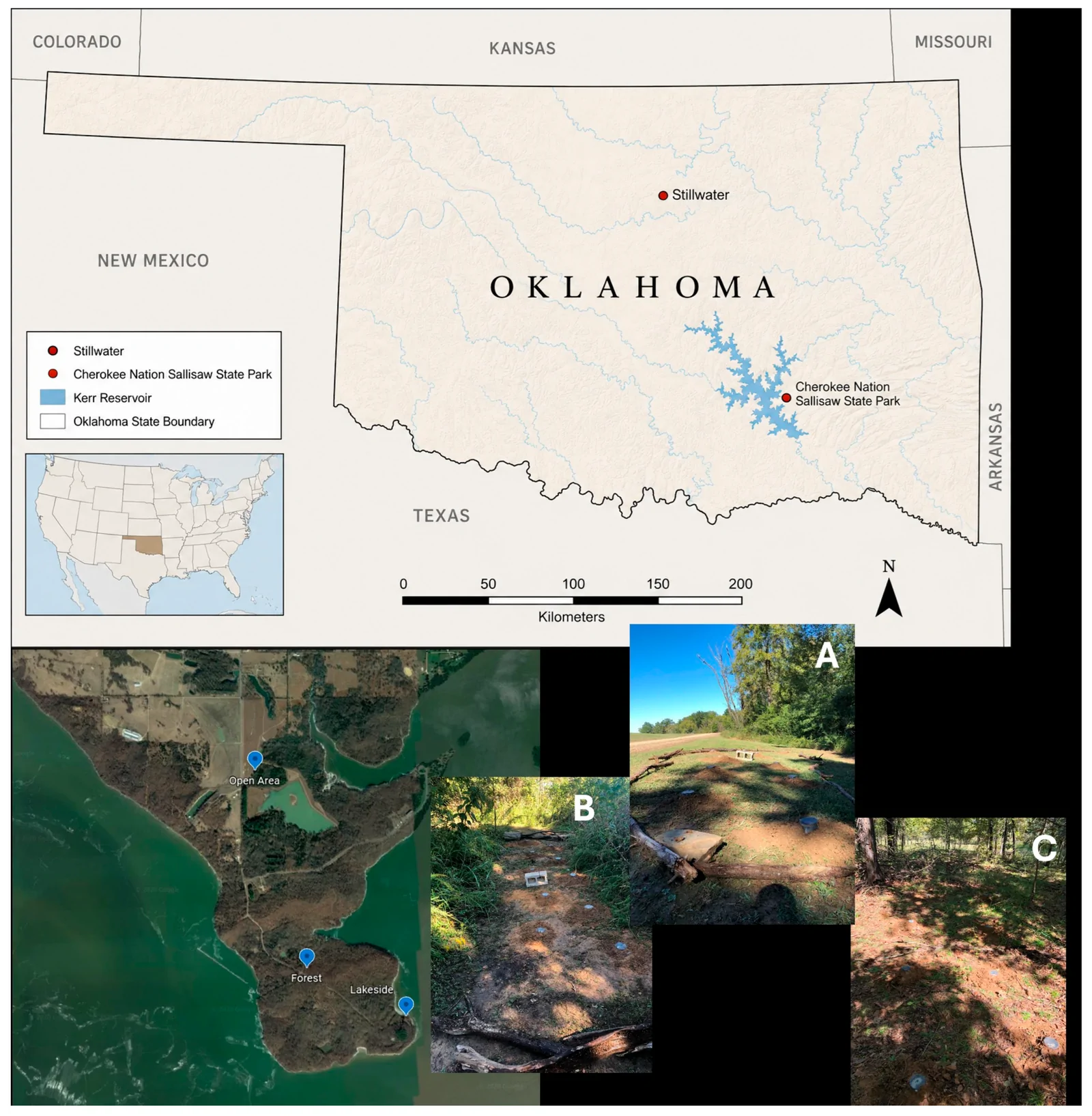
Study sites for *Nicrophorus americanus* overwintering studies. In 2019/2020, survival was tested in an grassland habitat (open area) (**A**), a forested environment (**B**) and a shore environment (**C**) at Cherokee Nation Sallisaw State Park. In 2023/2024 and 2024/2025, survival was tested at field plots near Stillwater, Oklahoma.

These experiments investigated the overwintering ecology of *N. americanus* in Oklahoma. A laboratory study tested the super-cooling point of adult *N. americanus* of different ages. Field studies were conducted in different locations using experimental designs to test specific questions regarding how environmental conditions influence overwintering survival and burial behavior. A habitat-based overwintering experiment was conducted in 2019/2020 in eastern Oklahoma to determine the survival of *N. americanus* in three distinct habitat types (Figure 1). Beetles were provisioned with a rat carcass. A two-year (2023/2024 and 2024/2025) overwintering experiment was conducted in Stillwater, Oklahoma, to compare the survival of un-provisioned *N. americanus* in tubes that restricted beetle burial depths (Figure 1). Although the experimental designs differed, all experiments involved placing adult beetles into controlled soil environments during autumn and recovering them in early spring to evaluate overwintering outcomes.

### 2.2. Super-Cooling Point Determination

A colony of *N. americanus* is maintained at Oklahoma State University [21]. Beetles originated from Camp Gruber, Muskogee County. Laboratory experiments were conducted to determine the SCP of nine *N. americanus* adults of different ages following the methods of Carrillo et al. [26]. Test beetles were aged as teneral (newly emerged), mature (midpoint of adult lifespan), and senescent (nearing the end of life), based on the work of McMurry et al. [21]. Prior to the experiment, individual beetles were placed into a freezer for 8 min for anesthetization prior to removal of the right middle leg at the coxa. Following leg removal, a constant thermocouple consisting of 30-gauge copper wire was inserted into the coxal opening. Temperature data were recorded every 0.2 s during the experiment using a TC-2000 thermocouple meter (Sable Systems International, Las Vegas, NV, USA) attached to the thermocouple. Each beetle was placed into a 50 mL polypropylene centrifuge tube (Corning Inc., Corning, NY, USA) partially submerged in a dry ice and 70% ethyl alcohol bath contained in a 500 mL glass beaker. The latent heat of crystallization was used to determine the super-cooling point. Pronotal size, beetle weight, and age [21] were collected prior to testing.

### 2.3. Study Sites

The habitat-based overwintering experiment was conducted at Cherokee Nation Sallisaw Creek State Park in eastern Oklahoma. The park is located approximately 12 km from Sallisaw and contains diverse ecological habitats including forests, open grasslands, and lakeside environments. Three habitat types were selected to evaluate how environmental conditions influence the overwintering survival of *N. americanus*. We tested survival in a habitat adjacent to a large water body and characterized by shrubs and small trees (lakeside); a habitat containing a dense tree canopy and a thick organic litter layer (forest); and a habitat with short cool-season grasses (grassland).

At each habitat, a HOBO data logger (Onset Computer Corporation, Bourne, MA, USA) with a thermocouple was fastened with a zip tie to a cinderblock at the plot center. The loggers recorded air temperature three times per day, at 8 h intervals. Precipitation and reference temperature data were acquired from the Sallisaw Mesonet station (www.mesonet.org, accessed on 1 April 2020). Burial-depth trials were conducted at the Oklahoma State University Botanic Garden, 2.25 km west of the Stillwater campus. In 2023, we used plant beds that had previously held alliums and clover; in 2024, we used Stillwater-grown blueberry plots located roughly 30 m away. Each bed had received topsoil the previous year, and mulch overlying the 2024 bed was removed before installation of overwintering tubes. Mesonet data from the Stillwater airport, located approximately 10 km from the trial, were used to determine the maximum, minimum, and mean air temperatures, along with daily precipitation.

### 2.4. Overwintering Experiments

Individual *N. americanus* used in the experiments were the first generation produced from wild adults and were between 30 and 50 days old. Laboratory beetles were maintained on a diet of mealworms and waxworms. Prior to placement in tubes, each beetle was sexed, measured for pronotal width, and weighed. Each tube contained one beetle. After placement, tubes remained undisturbed for the duration of the overwintering period.

Each beetle was housed in a high-density polyethylene tube perforated with 4 mm holes (Pentair Aquatic Eco-Systems), which allowed rainfall, soil moisture, and temperature change to reach the enclosed soil. All tubes were 10.16 cm in diameter, while the length depended on each experiment. For the effects of environment, all tubes were 55 cm long. Tube openings were closed with a soft metal mesh secured by a hose clamp, reinforced by a chicken wire screen epoxied to the mesh; together, these prevented escape and excluded scavengers. Tube tops stayed above the grade. Soil was standardized across experiments by mixing organic soil with commercially purchased topsoil, and tubes were backfilled to about 5 cm below the rim. For the habitat experiment, tubes were spaced approximately one meter apart, minimizing interactions between experimental units. For the habitat study, each beetle was provided with a rat carcass (180 g purchased previously frozen from Rodent Pro (rodentpro.com, accessed on 15 May 2020). For the burial depth experiment, tubes were 55 cm, 30 cm, or 15 cm in length and were placed approximately 20 cm apart. Each *N. americanus* was given two meal worms (*Tenebrio molitor*) and two wax worms (*Galleria mellonella*) upon introduction into the tube.

In the subsequent experiments to test the effects of burial depth, tubes were constructed as above but constructed to different lengths. In the 2023 burial depth experiment, five individuals were placed in 15 cm tubes (short), five in 30 cm tubes (medium), and ten in 55 cm tubes (long). For the same experiment in 2024, eight individuals were placed in short tubes, eight were placed in medium tubes and 12 were placed in long tubes.

At the conclusion of the overwintering period, the tubes were excavated to recover beetles, and survival was determined. Soil from each tube was incrementally removed and sifted thoroughly until the beetle was located. For each tube, survival status and burial depth, measured as the vertical distance from the soil surface to the first visible body segment of the beetle, were recorded.

### 2.5. Statistical Analysis

Data collected from all field experiments included survival status, burial depth, morphological measurements, and environmental variables. Statistical analyses were conducted to examine relationships between overwintering survival and factors including burial depth, temperature, precipitation, and beetle body size. Comparisons among treatments were conducted using regression analyses and nonparametric statistical tests, where appropriate. Soil moisture differences were evaluated using Mann–Whitney U tests. All analyses were performed using SigmaPlot 13.0 [27].

Daily temperature and rainfall were compared between the two overwintering seasons over a shared calendar window (1 October–9 February, the period covered by both years), and one outlier temperature record on 12 October 2023 was removed. The temperature was compared on matched calendar days by pairing each day-of-winter across seasons and testing the paired differences with a Wilcoxon signed-rank test. Because seasonal daily differences remain temporally autocorrelated, even after pairing, the nominal test was supplemented with a 7-day moving-block bootstrap (95% CI for the mean difference). Rainfall was assessed three ways: wet-day frequency (a wet/dry × year table tested with Fisher’s exact test); wet-day intensity (a Mann–Whitney U test on the non-zero daily amounts); and timing (the rainfall centroid and the 50-accumulation date, i.e., the day by which half the season’s rain had accumulated). All tests used a two-sided α = 0.05.

## 3. Results

Super-cooling points were tested using nine adult *N. americanus*. Beetles were sorted by age, with teneral beetles having been eclosed in December, mature beetles in October, and senescent beetles in September. Teneral beetles had slightly lower SCPs (−2.35 °C) than did mature (−1.48 °C) or senescent (−1.53 °C) individuals, though differences were not statistically significant (Kruskal–Wallis non-parametric ANOVA; Table 1). Senescent beetles were slightly smaller in terms of pronotal width (10.32 mm) and mass (1.16 g), which may have contributed to the differences (Table 1).

### 3.1. Habitat Experiment

Across the three habitats, 17 of 29 *N. americanus* survived the field overwintering trial. Survival was 50% in both the open and lakeside plots (open: 3 males, 2 females; lakeside: 2 males, 3 females). Seven out of nine forest beetles (5 males and 2 females) survived, while one beetle could not be located and was excluded from the analysis of survival. Its tube showed no openings, and no body parts indicating mortality were recovered.

Upon recovery, the mean burial depth of open-area survivors was 3.05 ± 2.03 cm, while the dead individuals were found at 7.11 ± 4.98 cm. Lakeside survivors were found deeper at 21.34 ± 5.71 cm, and the dead *N. americanus* were located near the surface at 1.02 ± 1.02 cm. Finally, in the forest, surviving beetles were found at 15.60 ± 7.57 cm, and the dead beetles were located near the surface at 1.27 ± 1.27 cm. Because collection took place in March, when some individuals had resumed activity and were attempting to escape, measured depths were highly variable, and statistical comparisons were not made. Across the experiment, surviving beetles were larger as measured by pronotal width than were those that died (10.52 ± 0.11 mm vs. 10.06 ± 0.14 mm; Student’s *t*-test, *t* = 2.622, df = 27, *p* = 0.014).

**Figure 2:**
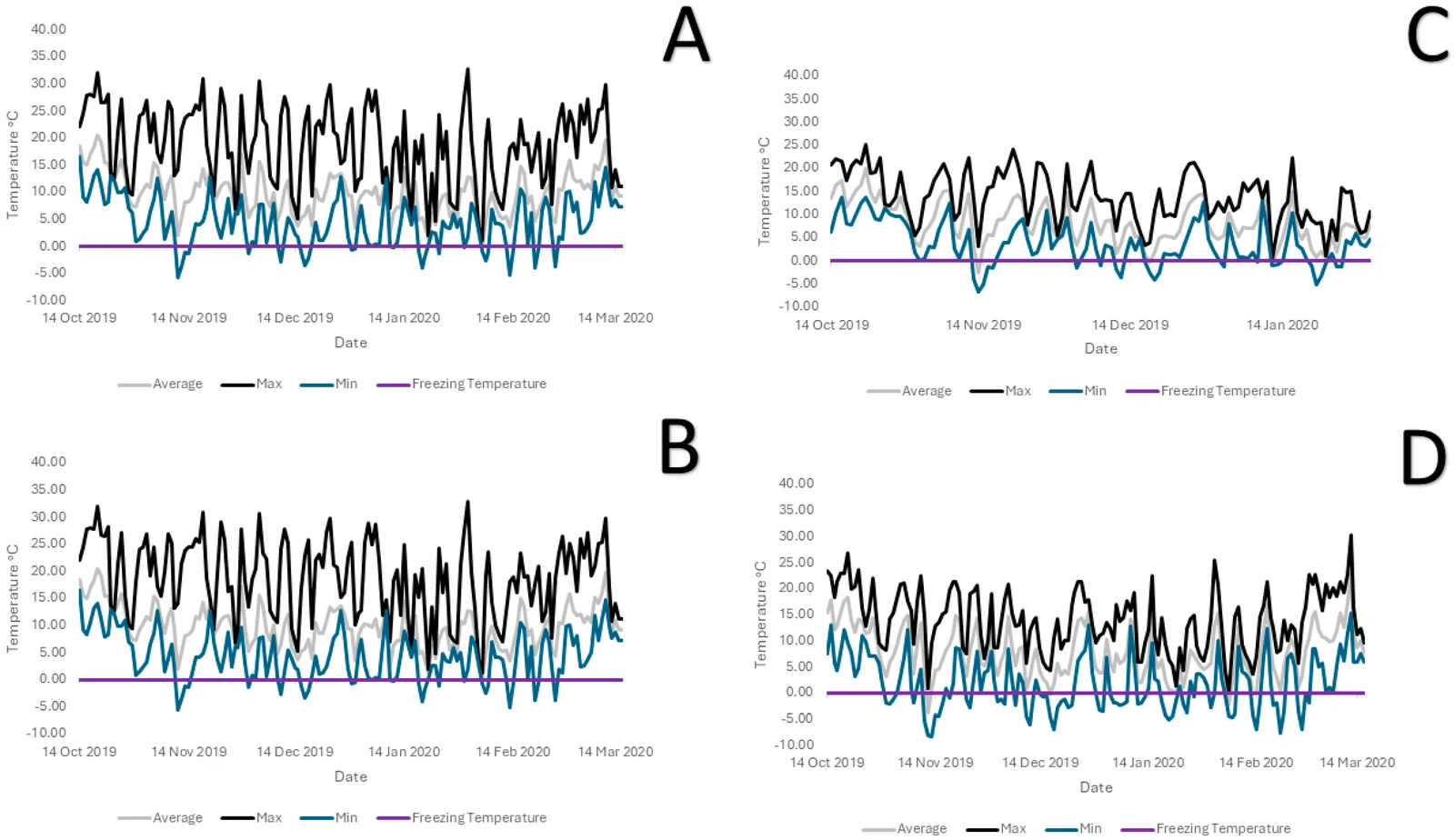
Maximum, minimum, and average temperatures recorded at (**A**) the grassland area, (**B**) the lakeside area, and (**C**) the forest area of the Cherokee Nation Sallisaw Creek State Park and (**D**) from the Sallisaw Mesonet Weather Station for October 2019 through March 2020.

Site records diverged from one another and from the Sallisaw station results. The grassland habitatsite (Figure 2A) ranged from 39.67 °C in October to −6.78 °C in November, with sub-zero minima on 43 out of 155 days (28%). The lakeside area (Figure 2B) peaked at 32.81 °C in February and bottomed out at −5.67 °C in November, with 26 of 155 days (17%) below 0 °C. In the forest, the logger failed in February 2020 (Figure 2C); over its 111 days of recording, the maximum was 25 °C in October, the minimum −6.82 °C in November, and 24 days (22%) fell below freezing.

The Mesonet station at Sallisaw (Figure 2D) recorded a high of 30.41 °C in March and a low of −8.22 °C in November, with sub-freezing minima on 68 of 155 days (44%). Across the same window, mean daily rainfall was 3.85 mm, the largest single-day total was 75.43 mm, and cumulative rainfall reached 549.91 mm.

### 3.2. Burial Depth Experiment

**Figure 3:**
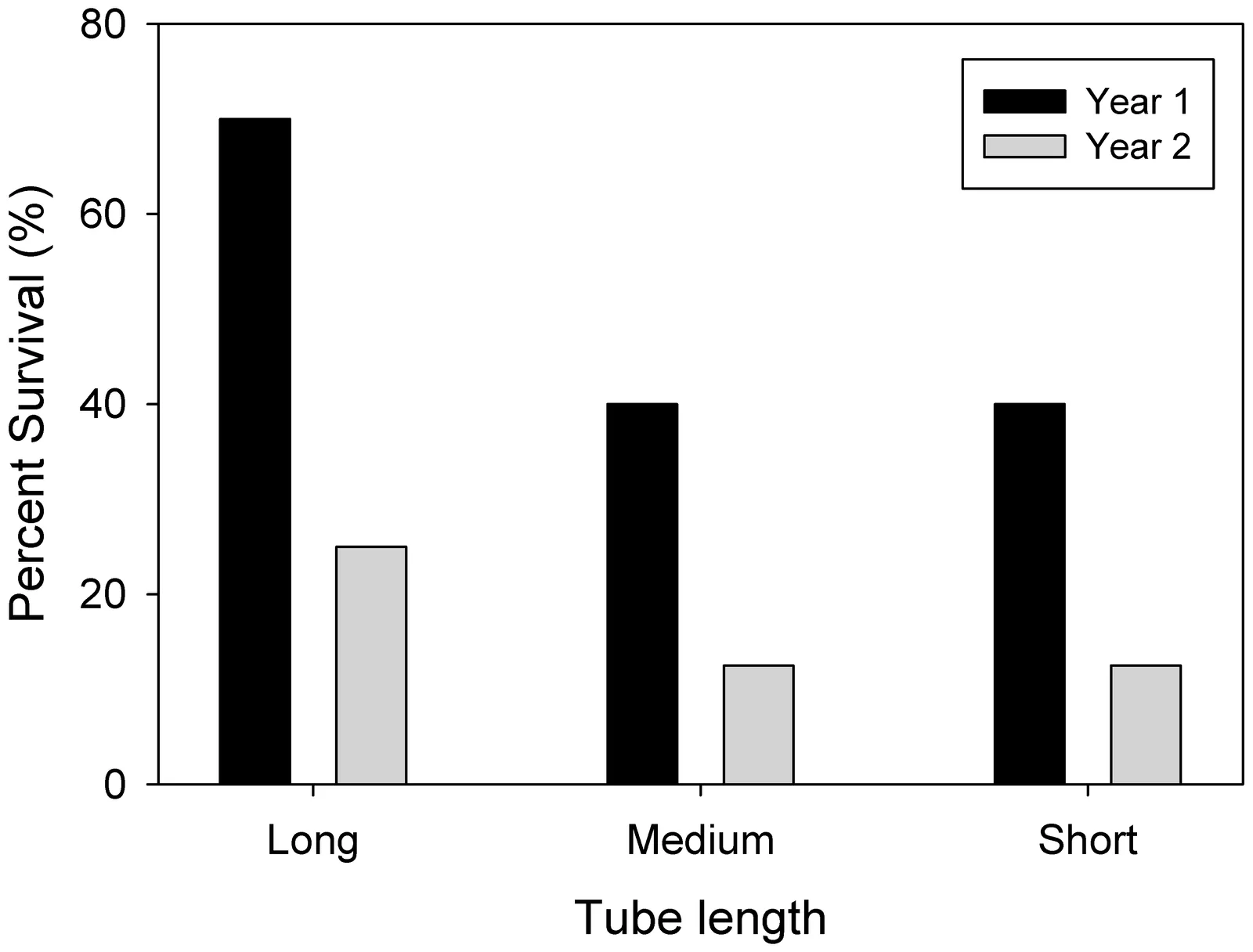
Percent survival of *Nicrophorus americanus* overwintering in 2023–24 and 2024–25. Survival was compared by maximum possible burial depth: long tubes (55 cm), medium tubes (30 cm) and short tubes (15 cm).

Maximum allowed depth of burial influenced overwinter survival for both years, as the longest tubes had the greatest survival rate (Figure 3). In 2023–2024, 11 of 20 tested *N. americanus* survived. Five *N. americanus* were tested in short- and medium-length tubes. Of those five per experiment length, one male and one female survived (40%). Ten *N. americanus* were placed in long tubes, with four males and three females surviving (70%). Survivors were larger than non-survivors, although the pronotal width (Student *t*-test, *t* = 0.305, df = 18, *p* = 0.34) did not differ significantly in either year (Student *t*-test, *t* = 0.486, df = 26, *p* = 0.651).

In 2024–2025, 5 of 28 tested *N. americanus* survived. A single male survived in each of the short and medium treatments compared with one male and two females surviving in the long tubes, i.e., 25% survival at 55 cm vs. 12.5% survival in both shorter tubes (Figure 3). Again, survivors and non-survivors did not differ in pronotal size (Student *t*-test, *p* = 0.288) or mass (Student *t*-test, *p* = 0.36).

### 3.3. Comparison Environmental Conditions Between Years

Matched-day temperature did not differ significantly between the two winters. Daily average temperature was 0.8 °C warmer in 2024–25, with an overnight minimum temperature difference of +0.3 °C, and was likewise non-significant (W = 3840, *p* = 0.81; block *p* = 0.76). Neither rainfall comparison was significant, yet wet days were less frequent in 2024–25 (20.5% vs. 25.0% of days). Wet-day intensity showed a higher mean in 2024–25, but this was driven by a few extreme events rather than by a pattern shift. The median wet-day total was slightly lower, and the distribution of amounts did not differ (U = 424, *p* = 0.75). Seasonal totals were similar overall (Table 2).

**Figure 4:**
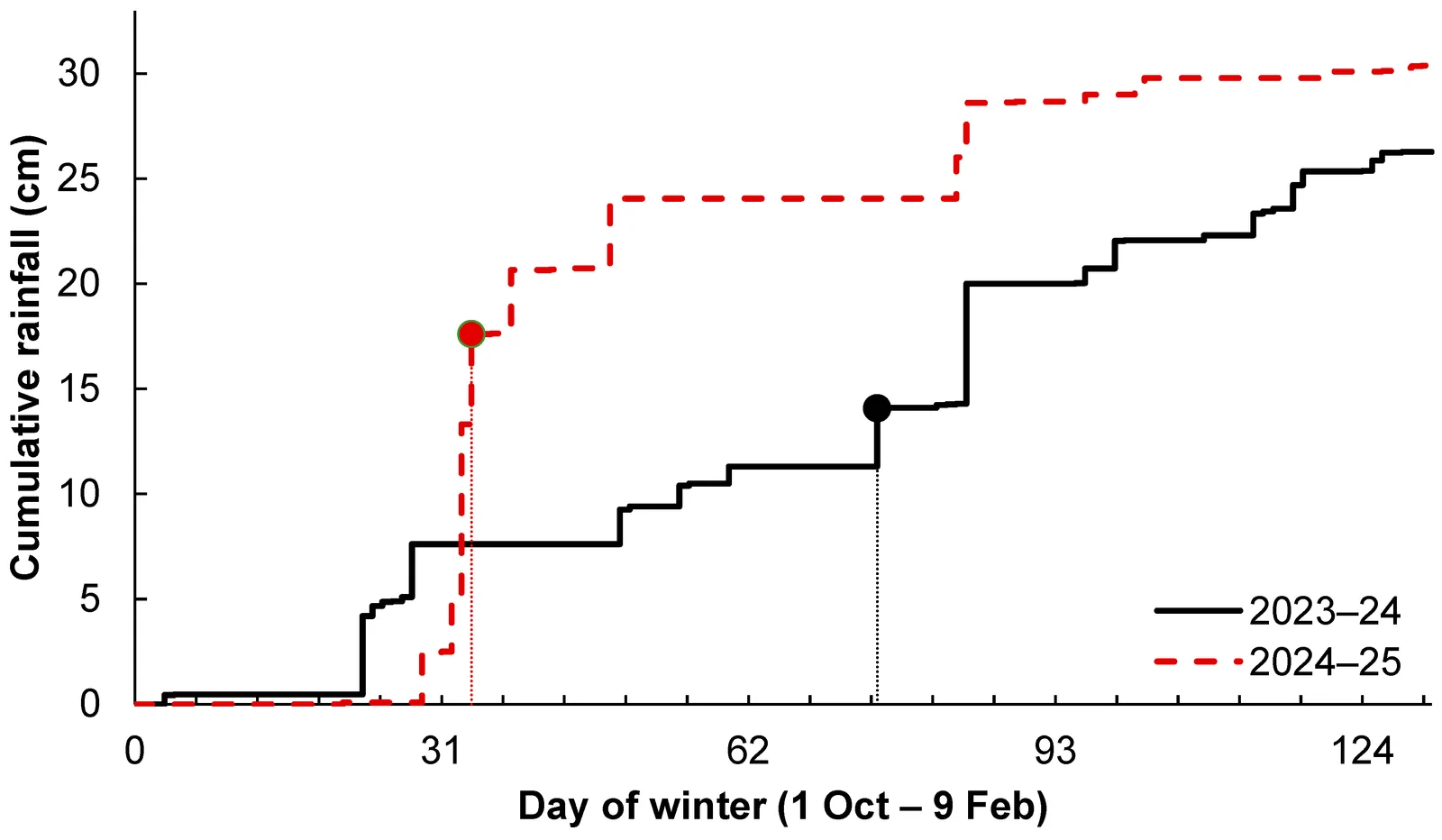
Cumulative rainfall (cm) by day of winter (1 October–9 February) for the 2023–24 (solid) and 2024–25 (dashed) overwintering seasons; markers show each season’s 50-rainfall date. The black dot indicates the 50% rainfall accumulation date for 2023–24, and the red dot indicates the 50% rainfall accumulation date for 2024–25.

Rainfall timing is the value for which the two winters diverged most clearly (Figure 4). In 2023–2024, rainfall was distributed through the season and peaked near midwinter. Its centroid falls on 8 December, and it did not reach its 50-accumulation point until 15 December, with 14.9 cm arriving across the December–February core of winter. In 2024–2025, rainfall occurred early, when approximately 71% of the seasonal total fell in November, with most of it in a single early-month event. Half the season’s rain had fallen by 4 November, and the centroid was 17 November; the remainder of winter was dry, with only 6.3 cm accumulating between December–February. The 50-accumulation date shifted roughly six weeks earlier in 2024–2025.

## 4. Discussion

### 4.1. Super-Cooling Point

Across the nine beetles tested, the mean SCP was −1.98° C (±0.2). Teneral individuals exhibited slightly lower SCPs, but body size alone did not explain the observed variation; rather, age, weight, and size may act together. In addition, environmental factors that prepare beetles to overwinter may influence the SCP. The observed differences in SCP among age classes should be interpreted cautiously because of the limited sample size and the low number of individuals within some age categories. Observations by Hoback and Conley [23] of movement of *N. orbicollis* during winter suggest that beetles remain active at temperatures near freezing and adjust their burial depth to stay below the frost line. The need to remain active is likely limiting cold tolerance strategies, such as production of glycerol, by *N. americanus*.

Wettlaufer et al. [28] measured lower lethal limits in *Nicrophorus sayi* Laporte, 1840 and *N. orbicollis* using extended cooling-bath exposures, followed by long recovery periods. Their results separated early- from late-emerging individuals and also varied with capture date, suggesting an age-effect. For *N. orbicollis*, which also occurs in Oklahoma, there was a 50% mortality below −2 °C for individuals collected in June and July (−2.9 ± 0.1 and −2.4 ± 0.01, respectively) [28]. Because immune responses of burying beetles differ with age and between natural and laboratory settings [29], laboratory lethal limits may also differ from field tolerance levels. The CTmin of −1.9 ± 1.9 °C reported for summer adults of the northern carrion beetle *Thanatophilus lapponicus* Herbst, 1793 [30] is closer to the value observed for *N. americanus* than to the SCP of −11.1 °C ± 0.7 °C reported for *Philonthus rotundicollis* (Ménétriés, 1832), a rove beetle that inhabits carpenter ant nests [31].

### 4.2. Overwintering Survival and the Importance of Microhabitat

The response of overwintering insects to climate change is still largely unknown [24,32]. Because *Nicrophorus* overwinter as adults, individuals can select a site, likely favoring conditions that improve survival [16]. Our experiments examined overwintering survival by confining beetles in tubes in specific habitats or by limiting the maximum depth beetles could burrow. Although tubes have exchange with the surrounding soils and allow beetles to choose depths, the tubes prevent beetles from choosing soil textures or from moving deeper to obtain moisture or laterally in search of microclimates affected by roots, rocks, or other characteristics. These differences may influence survival under natural field conditions, and future research should expand on these findings.

In the experiment testing survival of *N. americanus* in forest, grassland habitat and lakeside habitats, the same soil was used in all three areas, with all beetles housed in long tubes and all provided a rat carcass. Of 29 beetles, 17 survived (59%), 78% in forest plots, and 50% in each of the other two habitats. Shallow depths at recovery suggest beetles became active in the other areas, and survival may have been equal or greater. Repeated experiments at the gardens in Stillwater that tested the effect of maximum burial depth produced different results between years. These results strongly suggest that survival is more influenced by moisture availability than by habitat type or temperature fluctuations (Table 2).

Leasure and Hoback [20] found differences in habitat association among northern and southern *N. americanus* populations, which may relate to overwintering differences. Additionally, because environments are heterogeneous even at microscales and exhibit differences in composition, presence of roots, depth to ground water or rock, soil shading, etc., additional research should be conducted. Because *N. americanus* was historically found over a large range in the United States, it is classified as a habitat generalist, with disjunct populations occurring in southern and northern areas of the United States [20]. The current distribution supports the conclusion of local adaptations; however, the speed of these changes and genetic differences in the populations have not been discovered. With ongoing climate change affecting both temperatures and precipitation patterns, additional research on population differences is warranted and should be prioritized to conserve this federally designated threatened species.

Size at entry into winter has previously been linked to survival in the sexton beetle, *Nicrophorus investigator* Zetterstedt, 1824. Smith [17] found that larger Colorado individuals survived more often and suggested that this reflected the energy reserves accumulated beforehand. In our habitat experiment, survivors averaged measurements about 0.5 mm wider across the pronotum than did beetles that died. All rats appeared to have been consumed, but since tubes were not inspected between setup and retrieval, we cannot say whether decomposition rates varied by habitat. Notably, the carcasses were buried no more than a few millimeters, leaving them accessible through the tube openings to other insects such as phorid flies. If the rat was consumed by something other than the beetle, individual *N. americanus* would have relied on stored reserves, which could favor larger beetles over smaller ones.

Sexual maturity is reached roughly a month after emergence [21], and mature adults appear to withstand food deprivation better than do new adults [16,18], possibly because fat is deposited during adult activity. Because the new burying beetles do not reproduce until the following year, energy reserves gained prior to overwintering would be used for survival [16,25]. Alternatively, survival differences of mature adults may result from hardening of the exoskeleton in older individuals, which could contribute to resistance to desiccation [18,21].

A Nebraska study of *N. orbicollis* overwintering found a strong relationship between temperature and depth of burial, with individuals staying at or below the frostline and approaching the surface as temperatures increased [23]. The mean burial depth of *N. orbicollis* was 19.6 cm, which greatly exceeds the roughly 3.13 cm we measured for Oklahoma *N. americanus*. Again, this value was influenced by the March recovery date, when beetles had resumed activity and were attempting to leave the tubes. Heavy spring rains also likely influenced burial depths, as adults may have sought to escape the effects of flooded soils [19]. The lakeside treatment was likely more prone to flooding than was the open area, suggesting that high precipitation events and flooding could affect survival during overwintering.

### 4.3. Effects of Burial Depth

Allowed deeper burial improved survival during both years (Figure 3), suggesting that *N. americanus* seeks areas with deeper soils for overwintering. For winter survival, the size of *N. americanus* increased survival for the environmental test, but did not influence survival when the environment was uniform and the depth was constrained. Pipino [33] found pronotal width to be an important factor for survival, but other factors, including age, access to a carcass, and winter conditions appear to be more important for overwintering Oklahoma *N. americanus*.

For the burial depth experiment, the two years did not differ substantially in regards to minimum temperature or average temperature (Table 2). Rapid temperature swings occurred in both years, at comparable points in the season. However, both the amount of precipitation and its frequency differed substantially. In the first year, total precipitation during the experiment was 18.64 cm, while in year two, the total amount was 9.3 cm.

Total rainfall was higher in year one (18.6 cm vs. 9.6 cm), with precipitation distributed across more days. Despite differences in total amount, the timing of precipitation appeared to have the strongest influence on overwintering survival (Figure 3). The year-one maximum followed beetle placement (5.7 cm on 24 December 2023), whereas the year-two maximum preceded the start of the experiment (8.3 cm on 3 November 2024). Pre-placement saturation of this kind could have an impact, but is not likely associated with drowning because beetles were not confined to the soil during the heaviest precipitation in year 2 [19,23]. Despite difference in total amount, the timing of precipitation had the strongest influence on overwintering survival (Figure 3). Together, these data suggest that the analysis of weather based on the comparison of means may miss important factors that affect the interpretation of data.

### 4.4. Implications for Insect Conservation and Climate Change

Understanding the factors that influence overwintering survival is important for predicting how insect populations will respond to environmental change. Winter conditions are a key mortality factor for temperate insects, and small differences in overwinter survival can have large effects on population size in subsequent seasons [1,34]. Climate change is expected to alter winter conditions in many regions, potentially affecting overwintering insects in complex ways [24,34,35]. Warmer winters may reduce cold-related mortality for some species but may also increase the frequency of freeze–thaw cycles or disrupt diapause timing [34]. In some cases, warmer temperatures could lead to premature termination of diapause, leaving insects vulnerable to late winter cold events [24]. For insects that overwinter as adults, temperatures that are near freezing likely maximize survival by allowing conservation of nutrient stores during periods when foraging is not possible [35]. Warmer temperatures and dramatic fluctuation in temperature would both increase the use of these stores and may result in mortality associated with mass loss and starvation [35,36]. Changes in snow cover may also influence overwintering survival. Snow acts as an insulating layer that protects soil-dwelling insects near the surface from extreme cold temperatures [34,37]. Reduced snow cover may therefore expose overwintering insects to colder soil temperatures and increased mortality during winter [34,37].

Land-use change may further interact with climate effects by altering habitat structure and microclimate conditions. Agricultural intensification, soil disturbance, and removal of vegetation can reduce the availability of suitable overwintering habitats [38]. Conversely, conservation practices that maintain vegetation cover, preserve leaf litter, or protect natural habitats may enhance overwintering survival for many insect species [38].

## 5. Conclusions

Taken together, the Oklahoma experiments demonstrate that overwintering survival of *N. americanus* is influenced by a combination of behavioral, physiological, and environmental factors. Soil depth and habitat structure appear to play particularly important roles by modifying the microclimatic conditions experienced by overwintering individuals. Periodic precipitation is also a critical factor in beetle survival. The results highlight the importance of microhabitat heterogeneity in supporting insect populations during winter. Even small differences in soil depth, vegetation cover, or substrate composition may create microclimates that substantially affect survival. Consequently, maintaining diverse habitats with intact soil and litter layers may be critical for sustaining beetle populations in temperate ecosystems.

Future research should focus on quantifying the interactions between microclimate, physiological tolerance, and habitat characteristics to better understand how overwintering insects respond to environmental change. Such information will be essential for predicting population dynamics and developing conservation strategies for insects in changing landscapes.

## Figures and Tables

**Table 1:** Mean (±SE) super-cooling point, number of beetles tested, and size of *Nicrophorus americanus*.

	Number	SCP (°C) ^1^	Mass (g)	Pronotum (mm)
Senescent	2	−1.5 (±0.10)	1.2 (±0.41)	10.3 (±1.09)
Mature	2	−1.5 (±0.09)	1.2 (±0.02)	11.5 (±0.14)
Teneral	5	−2.4 (±0.25)	1.5 (±0.12)	11.2 (±0.41)
KW test ^1^		*p* = 0.238	*p* = 0.493	*p* = 0.466

^1^ SCP, super-cooling point; KW, Kruskal-Wallis non-parametric ANOVA.

**Table 2:** Between-year comparison of overwintering temperature and rainfall (2023–24 vs. 2024–25; common 1 October–9 February period). Temperatures compared on matched days by Wilcoxon signed-rank with an autocorrelation block-permutation *p*-value (Wilcoxon/block). Rainfall was compared with two metrics: wet-day frequency using Fisher’s exact test and wet-day intensity (median) by Mann–Whitney U test. The 50% rainfall accumulation is a descriptive statistic.

Comparison	2023–24	2024–25	Test	*p*
Daily mean temp	8.5° C	9.3° C	Wilcoxon/block	0.51/0.38
Daily minimum temp	2.0° C	2.4° C	Wilcoxon/block	0.81/0.76
Wet-day frequency	25.0%	20.5%	Fisher exact	0.46
Wet-day intensity (median)	0.23 cm	0.15 cm	Mann–Whitney	0.75
Rainfall 50% accumulation date	15 December	4 November	NA	—

## Data Availability

Data are available upon request.
